# Assessment of Phytotoxicity of Landfilled Waste and Foundry Dust Based on The Direct Test

**DOI:** 10.1007/s00128-022-03603-6

**Published:** 2022-09-08

**Authors:** Marta Bożym

**Affiliations:** Opole University of Technology, Prószkowska 76 Street, 45–758 Opole, Poland

**Keywords:** phytotoxicity, foundry waste, heavy metals, direct test, *Lepidium sativum* L

## Abstract

The article presents the results of phytotoxicity tests on foundry dust and landfilled waste. Currently, all this waste is being reused. The research has focused on phytotoxicity tests performed directly on the waste. Garden cress (*Lepidium sativum* L.) was used as the test plant. The germination test (GI) and the accumulation test were used to assess phytotoxicity. All dust types were highly phytotoxic to *L. sativum* L. in the direct test. The reason for this effect could be the low pH, the high level of heavy metals (HM), and probably the presence of organic pollutants (phenol, formaldehyde). The most phytotoxic was electric arc furnace dust (EAFD), classified as hazardous waste due to the high content of HM. The landfilled foundry waste (LFW) was characterised by moderate phytotoxicity in the direct test. The study confirmed the usefulness of the direct test in the phytotoxicity assessment of foundry waste with *L. sativum* L.

## Introduction

Landfilled industrial waste may have a negative impact on the environment and biota (Kicińska 2021). The toxicity depends on the composition, physical properties, and the leaching of the pollutants. Pollution of local soil or groundwaters around industrial plants or landfills may be an environmental problem (Kicińska 2019, 2020). Some industrial waste may be landfilled in heaps or piles, separated from the environment in order to minimise the infiltration of pollutants into the groundwater with geomembranes or natural materials (loams and clays) and to reduce dusting with biological reclamation using plants (Bożym 2018). Plants used for the biological reclamation of waste landfills should have a high tolerance for toxic substances (Remon et al. 2005). Most of the foundry waste is stored in industrial or municipal landfills; a small part is recovered (Bożym and Klojzy–Karczmarczyk 2021, Sabour et al. 2021). Due to the fact that the largest type of foundry waste by mass is spent foundry sand (SFS) (Dayton et al. 2010), many foundries regenerate SFS and re–use it for the production of castings, which reduces the consumption of raw materials. The demand for SFS is currently considerable, so it is profitable to recover waste, even landfilled waste (Bożym^2019^, ^2020^). SFS may be used as road aggregate in the construction industry and as a soil substitute in horticulture and agriculture (EPA Report 2014; Zhang et al. 2014). The latter application is popular in several countries, such as the USA, Argentina, Brazil, and the Republic of South Africa. The condition for such use of SFS is a low content of pollutants, including heavy metals (HM) (EPA Report 2014).

Foundry dust may be generated at various stages of the casting production, mainly during metal smelting, sand preparation, cleaning and knocking out of castings, and in the processes of dry sand regeneration (Bożym and Klojzy-Karczmarczyk 2020). The place of collecting dust samples affects its physical and chemical properties. For example, electric arc furnace dust (EAFD) is classified as hazardous waste because it may contain significant amounts of HM (Salihoglu et al. 2008; Mymrin et al. 2016; Bożym 2020). The preferred management of foundry dust is to reuse it, and it may be recycled in the foundry process or used for other purposes (Bożym and Klojzy–Karczmarczyk 2020). Dust with a high content of HM, which cannot be managed, should be solidified and then landfilled in properly prepared landfills. Due to the high costs of waste disposal, foundries independently manage all types of dust (Mymrin et al. 2016).

To assess the use of foundry waste, its quantity, physical/mechanical properties, binder content, composition, and toxicity, the content of pollutants and their leachability are taken into account (Dungan and Dees 2009; Dayton et al. 2010). Biotoxicity assessment may complement the physicochemical analysis of the foundry waste. Phytotoxicity tests may be useful to determine the impact of the waste on test species of plants and to calculate bioaccumulation factors. So far, foundry waste has not been assessed for its phytotoxicity. Some authors have studied the activity of microorganisms in soil substitutes based on investigated SFS (Dungan et al. 2006, 2009; Dungan and Dees 2009; Zhang et al. 2014). In phytotoxicity tests, the germination of seeds and elongation of the roots after 72h on leachate are usually evaluated. Both parameters are used to calculate the germination index (GI). Some authors observed that the root elongation is a more useful test than the germination test to assess the phytotoxic effect (Fuentes et al. 2004; Mitelut and Popa 2011; Kicińska and Wikar 2021). The root is more sensitive to toxins because it is directly exposed to the toxic effects of the contaminated solution (Fuentes et al. 2004).

Vegetative tests allow for a wider assessment of the phytotoxicity and accumulation of pollutants than germination tests (Jayasinghe 2012; Gyuricza et al. 2010) suggest that direct tests on the substrate are more useful than leachate tests in assessing phytotoxicity because plants depend directly on the substrate. For this reason, the contact of seeds with the substrate reflects the real conditions. However, for GI tests directly on a substrate, root germination and root elongation may be more difficult to evaluate because the roots may penetrate the substrate, and consequently, a visual assessment may be more complicated (Bożym et al. 2021). Selecting an appropriate species for the type of toxin and waste may be problematic because each of the species may be characterised by a different tolerance to contamination (Manas and De las Heras 2018). One of the species used to assess phytotoxicity is garden cress (*Lepidium sativum* L.). This species is used in the toxicity assessment of HM (Visioli et al. 2014; Masarovičová and Kráľová 2017). Garden cress is highly tolerant to salinity, drought, and high concentrations of HM and metalloids (Visioli et al. 2014; Masarovičová and Kráľová 2017; Praveen et al. 2017); for this reason, it is often used in phytoremediation (Dursun and Ayturan 2018; Das and Osborne 2018). The advantage of using cress in phytotoxicity tests is its rapid growth, common occurrence, the availability of its seeds, and the ease of analysis (Masarovičová and Kráľová 2017). *L. sativum* L. is used to assess the phytotoxicity of soils (Mekki and Sayadi 2017; Manas and De las Heras 2018), sewage sludge (Fuentes et al. 2006), composts (Aslam et al. 2008), or industrial waste (Bożym 2020; Bożym et al. 2021; Kicińska and Wikar 2021). In order to assess the accumulation of metals in the test plant, the Bioconcentration Factor (BCF) (Ashraf et al. 2011) or the transfer factor (TF) (Saha et al. 2017) are used. BCF can be calculated for all or part of a plant, e.g. for the roots (BCFr) or shoots (BCFs) (Marchiol et al. 2004; Kandziora–Ciupa et al. 2017). Some authors state that BCF is only concerned with the accumulation of metals in the root, while the bioaccumulation coefficient (BAC) is concerned with the metal content in the shoots (Amin et al. 2018). These factors are calculated as the ratio of the metal content in the part of the plant examined compared to the content in the substrate or leachate (extract).

The aim of this study was the evaluation of foundry waste phytotoxicity and HM accumulation by *L. sativum* on the basis of direct tests on the substrate. Moreover, the evaluation of the usefulness of direct testing in assessing the toxicity of foundry wastes was analysed.

## Methods and Materials

**Fig. 1:**
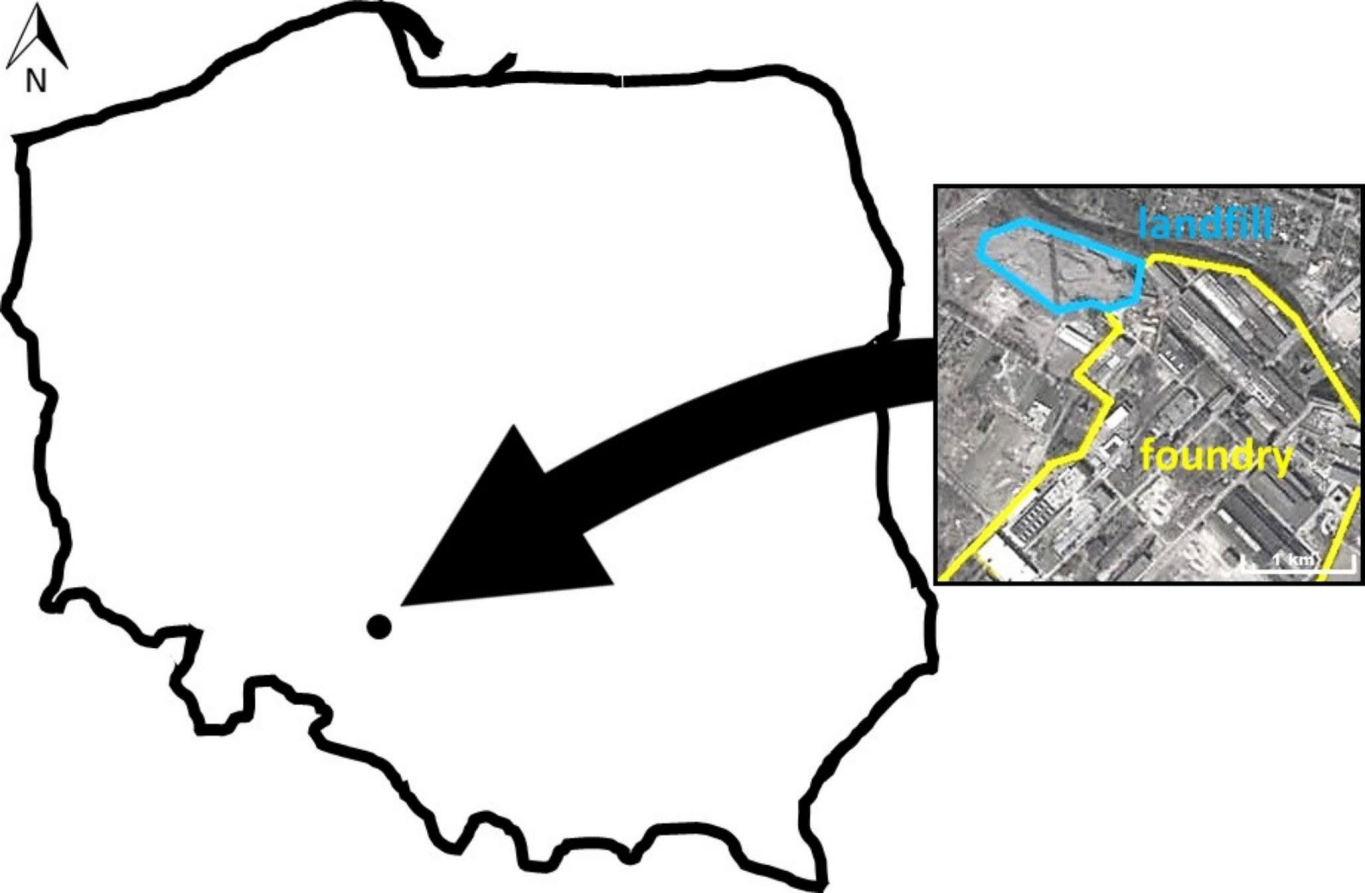
Location of the foundry, Opolskie Voivodeship, Poland

Samples of waste were collected from iron and steel Polish foundry (N 50^o^40^,^25.609; E 18^o^12^,^33.285) (Fig.1). For the production of foundry moulds, organic binders based on phenol–formaldehyde resins, and less frequently, bentonite, are used. Samples of the landfilled foundry waste (LFW) (n = 6) were taken from the landfill located next to the foundry. The samples were taken from six piles after prescreening the waste during the recovery process. The main component of this waste is SFS (approx. 80% wt.), slag (approx. 10% wt.), spent refractory materials (approx. 5% wt.), and others (dust, metalliferous inclusions). Primary samples were taken from several places on each pile to form an incremental sample. Primary samples (n = 5; approx. 5kg each) were reduced to laboratory samples by quartering to the volume of approx. 4–5kg. The second group of waste collected for phytotoxicity tests was dust from dust collectors located in various units of the foundry, i.e. regeneration (RD) (n = 2), transport (TD), shock grating (SGD) (n = 2), electric arc furnace (EAFD) (n = 2), and pneumatic blast cabinet (PBCD) (n = 10) dust collectors. All types of dust are used for various purposes, i.e. EAFD and SGD are substrates for the production of briquettes for foundries, while the other dust is used as inert material in closed mines or producing building materials. According to the Polish classification of waste, foundry waste based on SFS is classified as ‘*waste cores and moulds after the casting process’* (code 10 09 08), while dust is classified into two groups depending on the content of hazardous substances (code 10 09 09 and 10).

## Phytotoxicity Assessment and Accumulation Test

In this study, phytotoxicity tests directly on the waste as ‘contact tests’, were carried out. A germination index (GI) and an accumulation test were performed. For the tests, untreated *L. sativum* L. seeds of certified commercial material (No. PL–EKO–01) were used.

## Germination Index (GI) and Accumulation Test

The experiments, germination index (GI) and accumulation test for each waste were performed in triplicate. The GI test was based on the analysis of the number of germinated seeds and the degree of root elongation over 72 days. The seeds were sown directly on the waste substrate, 20 per Petri dish (fi 10mm). Previously, about 10.0 ± 0.5g of waste was introduced into the dishes and soaked in deionised water (approx. 10 ml) for 24h to dissolve the pollutants. The Petri dishes with seeds were covered and incubated in the dark at room temperature for 72h. A control (sand) was prepared in a similar way. The sand was previously processed: at 550^o^C and treated with HNO_3_ and deionised water. The content of pollution and LOI of control sand did not exceed LOQ (Bozym et al. 2021). The EC value of the leachate from the control sand did not exceed 10 µS/cm. The GI value was calculated from the relative seed germination (RSG, %) and relative root growth (RRG, %), in accordance with Zucconi’s methodology (Zucconi et al. 1985). Petri dishes filled with waste were used for the accumulation test, as with the GI test. The experiment was carried out for no longer than 7 days in order to eliminate the stressful effects due to crowding. The experiment was completed with the emergence of the cotyledons. The waste was previously soaked in deionised water (approx. 10 ml) for 24h. In the next stage, 1.0 ± 0.1g of *L. sativum* L. seeds were sown. The dishes with seeds were covered and incubated for 7 days at room temperature for 16h in the light and 8h in the dark (ST2BD Smart incubator, Pol–Eko–Aparatura SP). After 7 days, the colour of the cotyledons was assessed. Only the aerial part (shoots) biomass of *Lepidium sativum* L. was used for the study of HM accumulation because it was difficult to clean the roots from the dusty substrate. Root contamination may have affected the HM analysis. The bioconcentration factor (BCF), the ratio of HM concentration in the shoots to the total content in the substrate, was calculated. BCF > 1 indicates bioaccumulation of the metal by the plant (Marchiol et al. 2004; Kandziora–Ciupa et al. 2017).

## Sample Ureparation and Analysis

LFW samples were dried at room temperature, ground in a mortar and sieved through a 1mm sieve. Dust samples were not ground and sieved but dried the same as LFW samples and analysed. The evaluation of germination and root elongation was carried out with an accuracy of 1mm. Germination was found when the sprout was > 1mm. Waste, sand (control) and plant samples were mineralised with inorganic acids (PN–EN 13,656, EN 16,173) for the determination of HM (Cd, Pb, Cu, Zn, Ni, Cr, Mo, Co). The mineralisation was carried out in a microwave oven (Start D, Millestone). HM were determined by the method of flame atomic absorption spectrophotometry (FAAS) using a spectrophotometer Solaar 6M (Thermo) (US EPA Method 7000B, PN–ISO 8288). In the waste samples, the loss of ignition (LOI) at 550^o^C (PN–EN 15,169); electrical conductivity (EC) as an indicator of the salinity (1:5, m/V) (PN–ISO 11,265); and pH in H_2_O (PN–ISO 10,390) using pH–conductometer CPC 501 (Elmetron) was analysed.

## Statistics and Quality Control

The statistical analysis was carried out using the program Statistica ver. 13.3 (TIBCO StatSoft Inc., Poland). All samples were analysed in triplicate. Certified reference materials (CRM) for quality control such as *‘Metals in soil’ (SQC001, Merck), ‘Urban particulate matter’ (SRM 1648a, Sigma Aldrich), ‘Fine dust PM10–LIKE’ (ERM®–CZ120, IRMM), ‘Lichen (trace elements’ (BCR–482, IRMM)* were analysed. Heavy metals content recovery in CRM samples ranged from 90 to 110%. The limit of quantification (LOQ) for total metals content was Cd 0.2mg kg^− 1^ DM; Pb, Cu, Zn, Ni, Cr, Mo, Co 0.5mg kg^− 1^ DM; pH 0.1; LOI 0.1% wt.; EC 0.001 mS cm^− 1^. To determine significant differences between waste samples and control results, a one–way ANOVA (Bonferroni t–test) was used. Correlation between root elongation/dry mass of *L. sativum* L. and pH, EC, LOI and sum of HM were calculated using single and multiple regression with the Statistica software.

## Results and Discussion

**Fig. 2:**
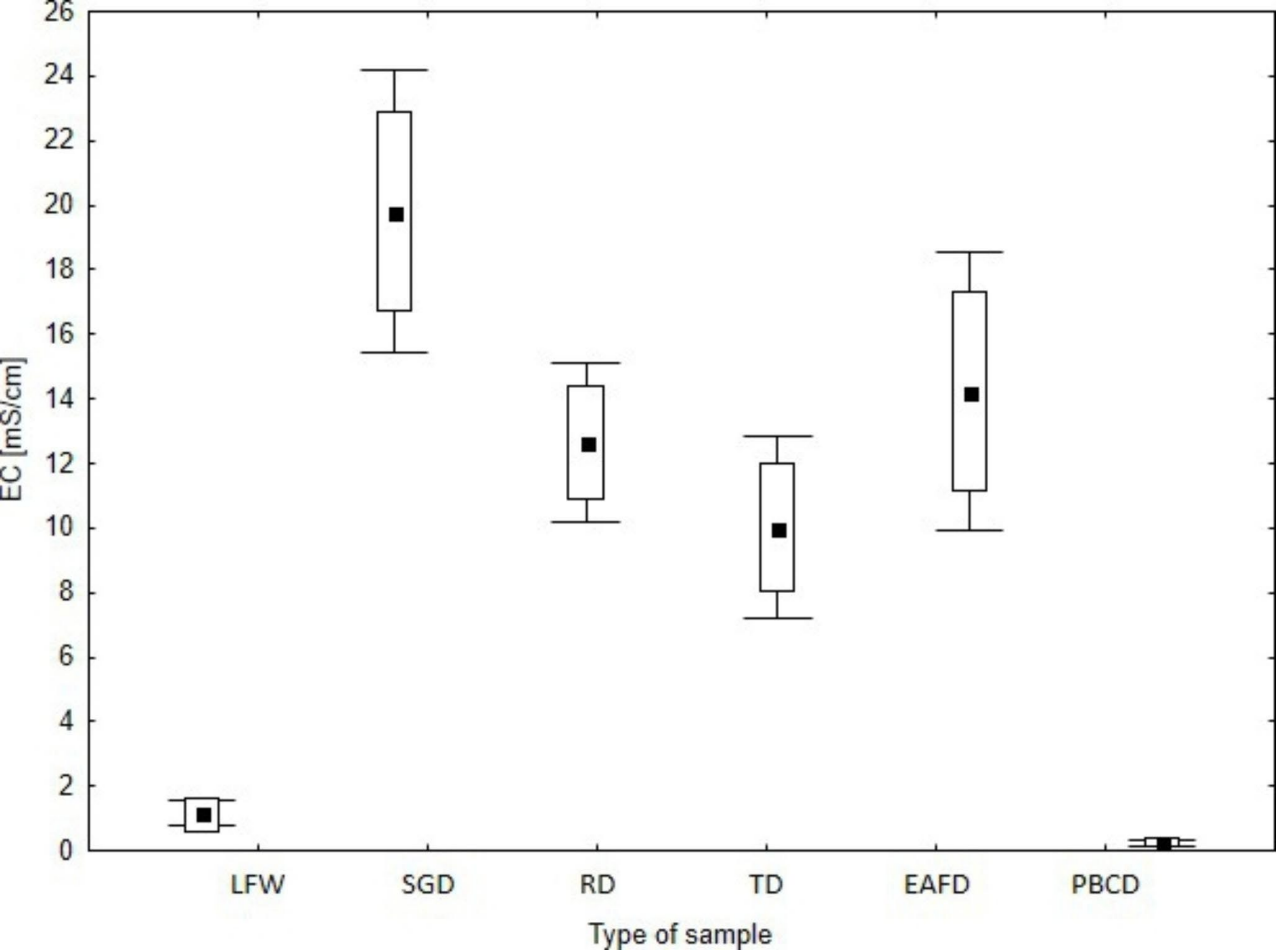
EC [mS cm^− 1^] of tested waste. The mean value is square point, edges of the boxes is min–max, the whiskers is SD, respectively

**Fig. 3:**
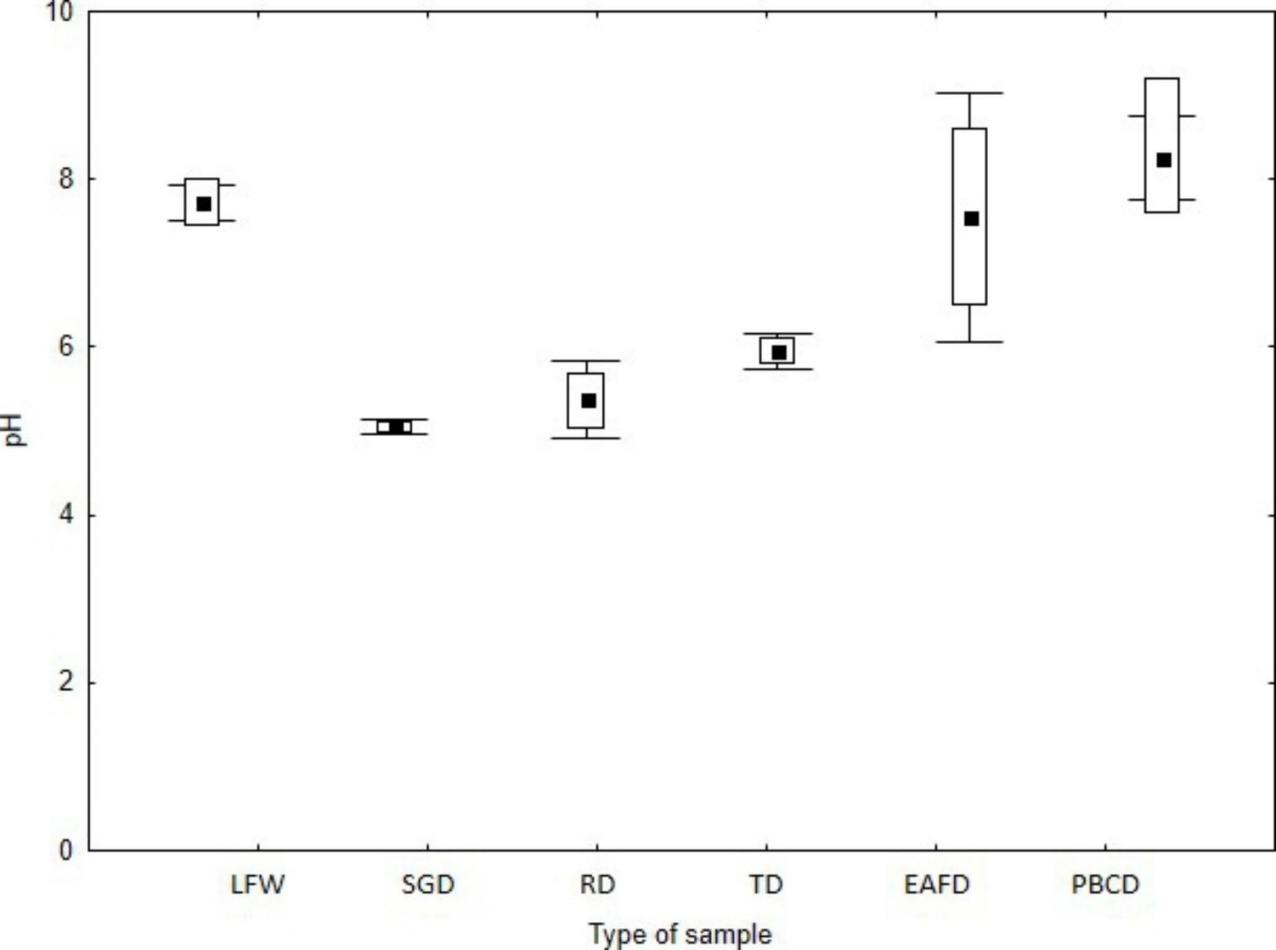
pH [H_2_O] of tested waste. The mean value is square point, edges of the boxes is min–max, the whiskers is SD, respectively

**Fig. 4:**
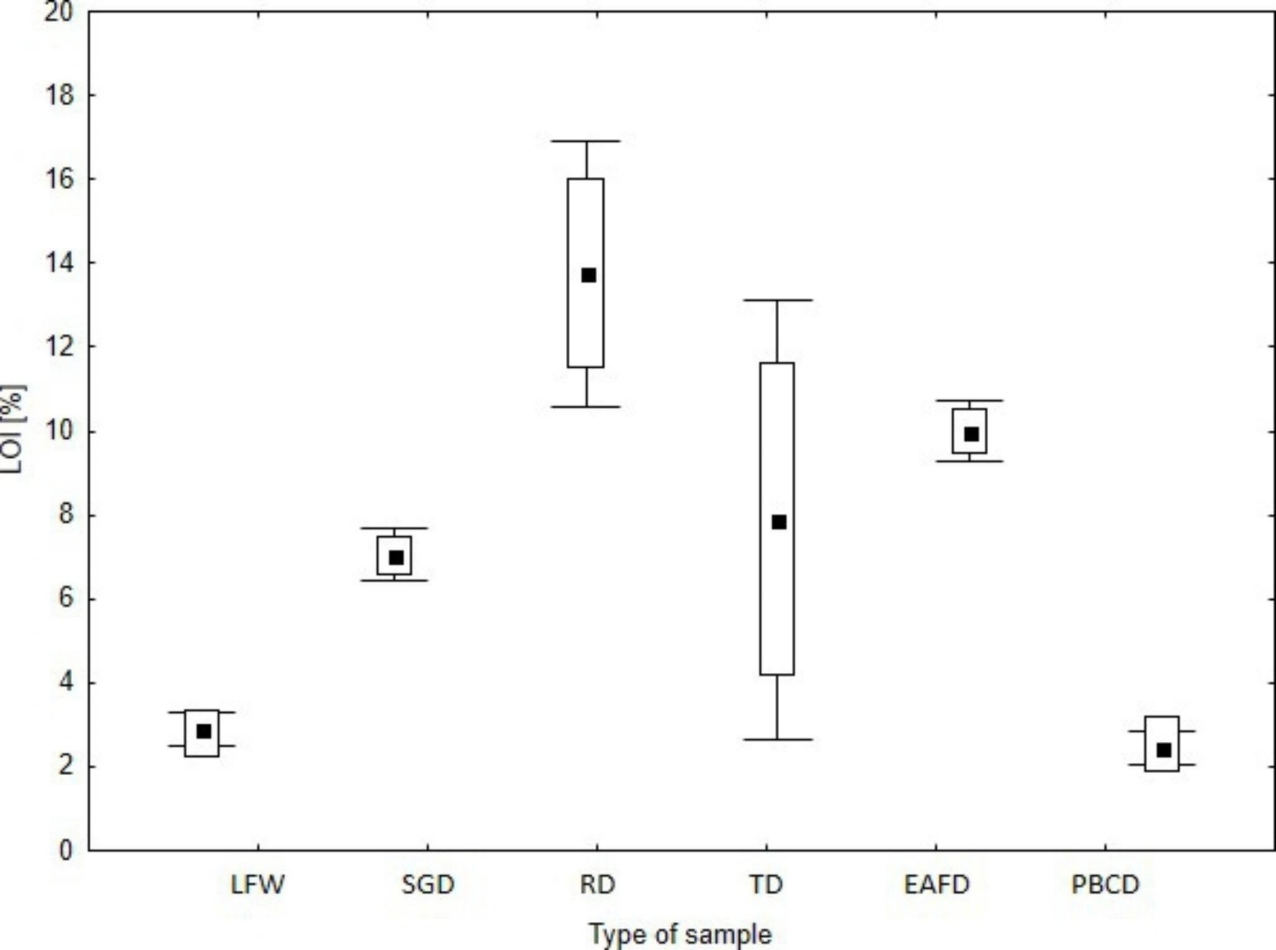
LOI [%] of tested waste. The mean value is square point, edges of the boxes is min–max, the whiskers is SD, respectively

The EC, pH and LOI of the tested waste are presented in Figs.2, 3 and 4. The tolerated soil EC value for plants is estimated at 2 mS cm^− 1^, which does not cause physiological changes. On the other hand, some species are tolerant to higher soil salinity, EC 2–16 mS cm^− 1^, and above this value (> 16 mS cm^− 1^) in the substrate, it causes plant death (Siuta 1980). The lowest EC value was found for dust samples PBCD (mean EC = 0.2 mS cm^− 1^) and FLW (mean EC = 1.2 mS cm^− 1^). The other types of dust were characterised by higher EC values (EC > 10 mS cm^− 1^), which may have a negative impact on the germination and vegetation of *L. sativum* L. The pH values of the tested waste varied within wide limits. The lowest pH was found for the dust samples, i.e. SGD (mean pH = 6.3), RD (mean pH = 5.4) and TD (mean pH = 6.0), respectively. A slightly alkaline pH was found for LFW (mean pH = 7.7), EAFD (mean pH = 7.5) and PBCD (mean pH = 8.2). The pH of foundry waste is influenced by the type of binder and less by the pH of the quartz sand (Dungan et al. 2006; Dayton et al. 2010; Holtzer et al. 2016). Excessively high or low pH may influence the biotoxicity of the waste (Phoungthong et al. 2016); for example, the toxicity of HM may increase at low pH (Emamverdian et al. 2015; Phoungthong et al. 2016; Seneviratne et al. 2017) found that phytotoxicity is the result of a combination of several factors that inhibit plant growth. For example, fluoride phytotoxicity increases at low pH (Stevens et al. 2000), while HM cations in solution may reduce the phytotoxicity of fluorides (Palmieri et al. 2014). The LOI value is an indicator of the presence of organic matter in the waste. Among the tested wastes, RD and EAFD samples demonstrated the highest LOI values, 13.8% and 10%, respectively. The dust from the regeneration section (RD) contained organic binder residues, hence its high LOI value (Bożym 2018), while EAFDs, similar to dust from thermal regeneration, may contain organic compounds from organic pollutants – such as adhesives, paints, or lubricants – contained in scrap, which may evaporate at high temperature during the metal melting process (Zanetti and Godio 2006; Salihoglu and Pinarli 2008). The lowest LOI values were determined for LFW (mean 2.9%) and PBCD (mean 2.5%). The organic matter (as LOI) of the tested waste may contain binder residues with phenol or formaldehyde, and because of that, it may be biotoxic (Bożym 2020).

**Fig. 5:**
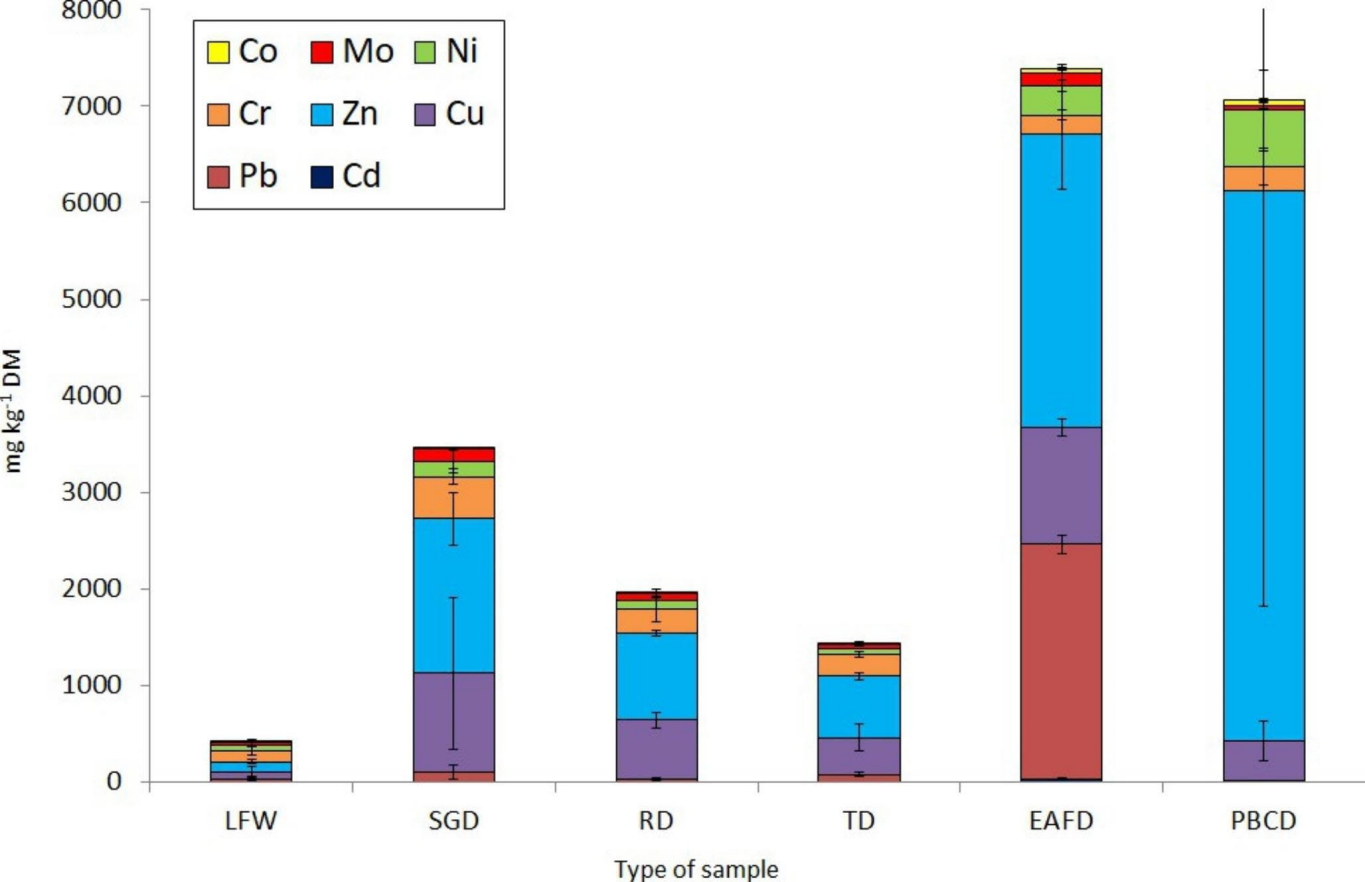
The content of HM [mg kg^− 1^ DM] in the tested foundry wastes (mean and SD values)

Figure5 shows the sum of HM (Co, Mo, Ni, Cr, Zn, Cu, Pb, and Cd) in the tested foundry waste. Among the HM, zinc characterised the highest content (23–81%), then copper (6–31%) and chromium (4–27%) for most wastes, and lead (33%) for EAFD. The highest content of all HM was found in EAFD dust. It is known that EAFDs are the most problematic waste in foundries due to the toxic content of HM and organic pollutants (Salihoglu and Pinarli 2008; Chirila and Ionescu Luca 2011; Mymrin et al. 2016; Bożym 2020). As Salihoglu and Pinarli (2008) found, metals such as Zn and Pb are very volatile at the temperature of molten steel and therefore accumulate in the furnace dust (Salihoglu and Pinarli 2008). Disposal of EAFD is problematic for foundries, as this dust may be generated in large quantities. It is estimated that in the typical operation of an electric arc furnace, about 2% of the input is converted into dust, and 10–20kg of EAFD are produced per 1 ton of castings (Chirila and Ionescu Luca 2011). Worldwide, foundries produce about 8million tons of EAFD annually; in the USA, it is about 0.7million tons, and in Europe, up to 1million tons (Chirila and Ionescu Luca 2011). HM may be recovered from the EAFD, but the cost–effectiveness of the process depends on the amount of metal in the dust (Li et al. 2010). In the current study, a large amount of HM, especially Zn, was also found in the dust from the shot blasting section (PBCD). Moreover, PBCDs were characterised by their high Ni and Cu concentrations. The lowest metal content of all tested dust samples was found in RD and TD, i.e. dust from the regeneration and transport units. On the other hand, the lowest HM content of all waste samples was found in the LFW. The LFW consisted mainly of SFS; therefore, this waste may be used for the production of road aggregates or other applications. This application is conditioned by the low leaching of pollutants (EPA Report 2014, Bożym 2020).

**Fig. 6:**
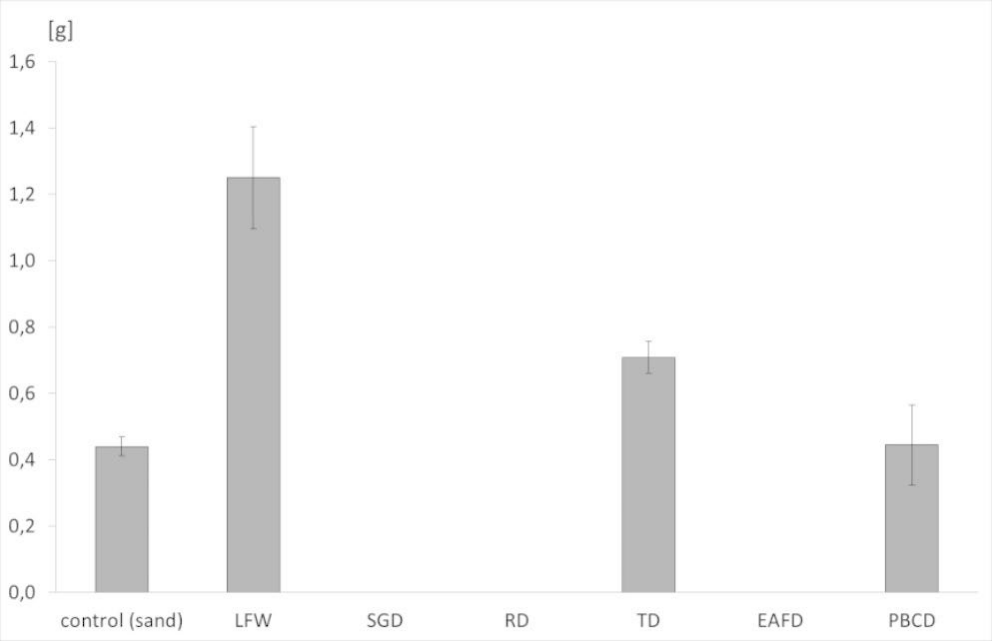
Dry mass [g DM] of shoots of *L. sativum* L. No plants have grown on the SGD, RD and EAFD substrate

According to Phoungthong et al. (2016), an excess of HM in the substrate causes stress in plants and leads to reduced germination, delayed growth, and leaf chlorosis. For this reason, the condition of *L. sativum* L. (cotyledon colour, shoot length, and biomass) was additionally assessed during the accumulation test. Blackening of the seed coat of *L. sativum* L. from the EAFD, SGD, and RD substrates was found. The same effect was observed in previous studies on leachate from foundry waste (Bożym 2020). Presumably, the cause of this effect was the high iron concentration and the low pH of the leachate. Mossor–Pietraszewska (2001) noted that Al inhibited root growth and darkened them, while Emamverdian et al. (2015) found blackened plants with a high Mn content in the substrate. However, in other studies with *L. sativum* L. growing on slag from copper and zinc smelters with high concentrations of Zn, Cu, and Pb in the substrate, this effect was not found (Bożym et al. 2021). In the current research, *L. sativum* L. sprouts died after 2–3 days of the accumulation test on EAFD, SGD, and RD substrates. This effect was not observed in previous studies for the leachate of those wastes (Bożym 2020). The best-coloured cotyledon of *L. sativum* L. was found in the LFW and control groups. On the other hand, the cotyledons of *L. sativum* L. of the control, LFW, TD, and PBCD groups were coloured well, indicating no negative effect of those substrates on chlorophyll. It is known that increased accumulation of HM in the aerial parts may cause leaf chlorosis, a reduction in yield, leaf area, relative growth rate, and assimilation rate (Keser 2013; Emamverdian et al. 2015; Masarovičová and Kráľová 2017). On the other hand, *L. sativum* L. is resistant to negative environmental factors, including HM (Dursun et al. 2018), and it is used as a bioindicator of the soil contamination from HM (Seneviratne et al. 2017). In addition to the visual assessment of the condition of the plants, a biomass analysis was performed. The aerial parts of *L. sativum* L. from the LFW, TD, and PBCD substrates and the control group were collected. The roots were not analysed due to the potential for contamination by the substrate, especially with dust, which could have affected the results. Figure6 presents the dry mass of *L. sativum* L. shoots obtained during the 7–day accumulation test. The results were compared with the control (sand). No plants were taken from the SGD, RD, and EAFD substrates. On the basis of the results, it may be stated that the biomass of *L. sativum* L. was significantly higher from the LFW substrate than in the control (p ≤ 0.05), which may indicate that LFW stimulated the growth of *L. sativum* L. This is confirmed by the results of the GI test. The reason for this effect could be the low total content and leachability of HM and also the neutral pH of LFW compared to the foundry dust samples (Bożym 2020). Moreover, the higher biomass of *L. sativum* growing on the TD substrate compared to the control (p ≤ 0.05) was also found. The probable reason for this effect was the low leachability of pollutants, the presence of macronutrients and the low EC of these wastes, which was confirmed in previous studies (Bozym 2020). Keser (2013) did not observe a negative effect of HM from wastewater on the biomass of *L. sativum* L., which explains the high resistance of this species.

Based on the bioconcentration index (BCF) (Table1), no metal accumulation by *L. sativum* L. in any substrate was found (BCF < 1.0), which indicates no ability for *L. sativum* L to bioconcentrate metals from foundry wastes. Slightly higher BCF values were calculated for Cu and Zn in LFW, Zn in TD, and Cd and Pb in PBCD. This effect was probably influenced by the short period of the experiment and the low percentage of mobile forms of metals in the waste. It is well known that the phytoaccumulation of HM from waste and soil depends on the total content, leachability, pH, and metal interactions in the substrate (Keser 2013; Seneviratne et al. 2017).

**Table 1:** Bioconcentration factors (BCF) in shoots of *L. sativum* L

metal	LFW	SGD	RD	TD	EAFD	PBCD
Cd	<LOQ	nd	nd	0.19	nd	0.44
Pb	0.07	nd	nd	0.18	nd	0.63
Cu	0.27	nd	nd	0.05	nd	0.08
Zn	0.67	nd	nd	0.28	nd	0.03
Cr	0.06	nd	nd	0.01	nd	0.02
Ni	0.02	nd	nd	0.01	nd	0.02
Mo	0.02	nd	nd	0.03	nd	0.08
Co	0.05	nd	nd	0.05	nd	0.03

nd – no data (no plants to analyze)

<LOQ - the metal content of the substrate or plants was below the limit of quantification.

## Germination Index

**Fig. 7:**
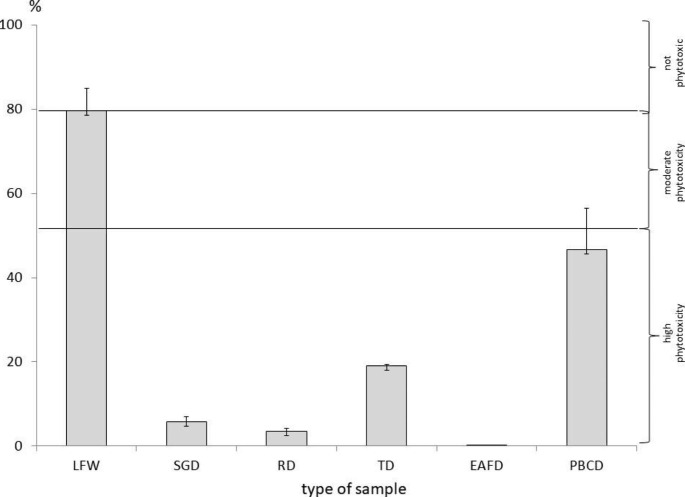
Germination index (GI) [%] calculated to direct test

The GI was calculated from the values for *L. sativum* L. germination and root elongation in the direct test on the substrate compared to the control (sand). Figure7 shows the GI value for *L. sativum* L. in the direct test. Additionally, the ranges of the inhibitory/stimulating action of waste on plants, according to Zucconi et al. (1985), were presented. A GI value of < 50% indicates high phytotoxicity, 50–80% indicates medium phytotoxicity, 80–100% no phytotoxicity, and > 100% suggests a stimulating effect on the plant growth.

Seeds from some substrates (EAFD, SGD, and RD) had blackened in the GI test, similar to the effect in the accumulation test. The *L. sativum* L. growing on these substrates was also characterised by shortened and blackened root tips. It is known that HM and the pH of the substrates have an impact on the root conditions and elongation (Emamverdian et al. 2015; Phoungthong et al.2016), such as with other toxic substances, e.g. phenols, cyanides, salinity, fluorides, ammonia, (Palmieri et al. 2014; Manas and De las Heras 2018). The contact test showed that LFW were characterised by moderate or no phytotoxicity; the dust samples, SGD, TD, RD, and PBCD, demonstrated high phytotoxicity (Fig.7). EAFD was characterised by the highest phytotoxicity among all waste samples in both the leachate and direct tests. The cause of the high phytotoxicity of this waste for *L. sativum* L. could be high total HM content and high salinity of the leachate (Bożym 2020). Other authors confirm the high toxicity of EAFD due to the high content of heavy metals and organic pollutants as well as the high leachable of those substances (Salihoglu and Pinarli 2008; Li et al. 2010; Chirila and Ionescu Luca 2011; Mymrin et al. 2016); however, no phytotoxicological studies have been conducted for these types of dust. In a previous study, leachate from all foundry waste was less phytotoxic than the substrate in a direct test (Bożym 2020). An additional phytotoxic factor could have been the low pH of some substrates and the presence of organic pollutants, i.e. phenol and formaldehyde. In other studies, low or no phytotoxicity was found in the leachate from foundry waste; in addition, some leachates had a stimulating effect on *L. sativum* L. (Bożym 2020). However, for hazardous industrial wastes contaminated with HM, higher phytotoxicity of this waste in the direct test than in the leachate test was found (Bożym et al. 2021).

## Correlation

On the basis of the single correlation analysis, statistically significant correlations were found for the dry mass of *L. sativum* L. and EC (r = − 0.79), pH (r = 0.56), LOI (r = − 0.77) between the root growth and EC (r = − 0.56), LOI (r = − 0.49), and the sum of HM in the medium (r = − 0.46) (α = 0.05). On the basis of the multiple correlation analysis, statistically significant correlation coefficients were found for the biomass of *L. sativum* L. and for the root growth and EC, with the sum of HM and LOI ranging from r = − 0.36 to r=–0.54 (α = 0.05). The correlation analysis shows that the inhibitory effect on *L. sativum* L. biomass is influenced by the salinity (EC) of the substrates and the content of the organic matter (LOI), the source of which are organic binders. A positive correlation was found between the pH of the substrate and the *L. sativum* L. biomass, which may be due to the reduced toxicity of HM at higher pH.

## Conclusion

Foundry dust was characterised by high phytotoxicity in the direct test, especially EAFDs, which contained the highest concentration of HM. Despite that the PBCDs were also characterised by a high content of HM, they were not as highly phytotoxic as the other types of dust. Moderate phytotoxicity was found for LFW. For this waste (LFW), an increased *L. sativum* L. biomass compared to the control was also found, which may suggest its stimulating effect. However, this has not been confirmed in GI tests. No ability for bioconcentration of HM by *L. sativum* L. in the contact test with foundry wastes was found. The probable cause of this effect was the short period of the experiment (7 days) and the high concentration of plants in the Petri dishes. Correlation analysis showed a negative effect of salinity (EC), organic matter (LOI) and HM on the roots elongation and biomass of *L. sativum* L. This study confirmed the usefulness of *L. sativum* L. for the assessment of the phytotoxicity of foundry waste. On the basis of the results of other authors and own research, it was additionally found that the tests directly on the substrate may give higher phytotoxicity than the leachate tests.
